# Supplementary material for: Coaching visits and supportive supervision for primary care facilities to improve malaria service data quality in Ghana: An intervention case study
Source: PLOS Glob Public Health. 2025 Jun 6;5(6):e0003649. doi: 10.1371/journal.pgph.0003649 (PMC12143497; doi:10.1371/journal.pgph.0003649)
Supplement: S1 Table — (DOCX) [file pgph.0003649.s001.docx]

**S1 Table. Variables used for error computations, with definitions (Source: National HMIS SOP, 2020)**

| **No.** | **VARIABLE** | **DEFINITION** |
| --- | --- | --- |
| 1 | Uncomplicated Malaria Suspected | All outpatient department new cases that the clinician suspects to be malaria; including pregnant women |
| 2 | Uncomplicated Malaria Suspected Tested | Total number of suspected cases of malaria tested for malaria parasites (both rapid diagnostic tests (RDTs) and Microscopy); including pregnant women |
| 3 | Uncomplicated Malaria Tested Positive | Total number of suspected cases of malaria that tested positive for malaria parasites (using both RDTs and microscopy); including pregnant women |
| 4. | OPD attendants treated with Antimalarials | Confirmed cases treated with any antimalarial drug according to national guidelines |
| 5. | OPD attendants treated with ACTs | Confirmed cases treated with artemisinin-containing combination therapy (ACT) according to national guidelines |
| 6. | Number of suspected uncomplicated malaria cases tested for malaria parasites using RDT | Total number of suspected cases of malaria tested for malaria parasites (using RDT); including pregnant women |
| 7. | Number of suspected uncomplicated malaria cases tested for malaria parasites using Microscopy | Total number of suspected cases of malaria tested for malaria parasites (using microscopy); including pregnant women |
| 8. | Number of suspected uncomplicated malaria cases tested positive using RDT | Total number of suspected cases of malaria tested positive for malaria parasites (using RDT); including pregnant women |
| 9. | Number of suspected uncomplicated malaria cases tested positive using microscopy | Total number of suspected cases of malaria tested positive for malaria parasites (using microscopy); including pregnant women |
| 10. | Number of ANC Registrants | Number of pregnant women reporting for antenatal care (ANC) for the first time to any health facility with their current pregnancy. |
| 11. | Number of pregnant women receiving IPTp1 | Number of pregnant women given their first dose of sulfadoxine-pyrimethamine (SP) at ANC |
| 12. | Number of pregnant women receiving IPTp2 | Number of pregnant women given their second dose of SP at ANC |
| 13. | Number of pregnant women receiving IPTp3 | Number of pregnant women given their third dose of SP at ANC |
| 14. | Number of pregnant women receiving IPTp4 | Number of pregnant women given their fourth dose of SP at ANC |
| 15. | Number of pregnant women receiving IPTp5 | Number of pregnant women given their fifth dose of SP at ANC |
