# Supplementary material for: Coaching visits and supportive supervision for primary care facilities to improve malaria service data quality in Ghana: An intervention case study
Source: PLOS Glob Public Health. 2025 Jun 6;5(6):e0003649. doi: 10.1371/journal.pgph.0003649 (PMC12143497; doi:10.1371/journal.pgph.0003649)
Supplement: Attachment — Submitted filename: PLOS Global Public Health Response to Reviewers.docx [file pgph.0003649.s003.docx]

We thank the reviewer for the careful attention to the methodological detail and flow of the manuscript and the helpful suggestions for revision. We attempted to make all the changes recommended by the reviewer and believe the reorganized methods section and improved description of the data coaching timeline and components included in the evaluation are now much clearer.

| **Reviewer comment** | **Action taken/response** |
| --- | --- |
| - A variety of terms are used to describe the various stages of the study and data collection: “baseline”, “post-intervention”, “first follow-up”, “second follow-up”, “midline”, “endline”, “post-endline”. To improve clarity, I strongly recommend using consistent terms to describe each period when outcomes are assessed, as well as when the coaching intervention takes place, and a statement in the methods clearly defining these terms. A timeline figure (or adapting figure 1) may also be useful if the scheme is complex. | We understand and agree that the multiple evaluation data sources and measurement points caused confusion. We streamlined terms and revised Figure 1 to reflect the difference between the intervention and the evaluation as well as the different evaluation components arranged spatially in a timeline model. |
| - The authors refer to a single data coaching visit, but multiple follow-up visits. Presumably these additional visits also comprise feedback being given, so should these be considered as coaching visits as well? | We originally conceptualized the intervention as a coaching visit with follow-up, but the reviewer rightly notes that the follow-up visits served the purposes of reinforcing skills/knowledge, assessing skills/knowledge, and orienting new staff, in addition to following the Plan-Do-Study-Act Cycle. We revised the text (and Figure 1) to consider all three visits as coaching visits. |
| - The structure of the manuscript could be improved. While I appreciate that this paper is presented as a case study, the attempt to conduct an evalaution of the coaching approach rather than simply report what was done means there is a need to clearly articulate some key details that would be part of a normal methods section. Perhaps a methods section with sub-titles including: study site / facility selection, intervention implementation/description/timeline, evaluation design, outcome indicators, analysis approach would help to improve flow. | We appreciate this observation; the reviewer is correct that a case study format was insufficient to describe the intervention as well as the evaluation. For clarity, we reorganized the text to separately describe the coaching intervention and the methods for the evaluation of the intervention. We reorganized the methods section and added subtitles as suggested. |
| - Line 90. I’m not familiar with “regional holistic assessments”. If this mechanism is particularly important or innovative, it could be helpful to add either a brief description or reference. | Here, we refer to the routine use in Ghana of a standardized tool used for health systems performance assessment called a “holistic assessment tool”. This tool provides a framework for assessing the health sector comprehensively and holistically. It uses various instruments to determine the sector's progress towards achieving set objectives. We changed the description to the more generic “health systems performance assessment” and added a citation for Ghana’s most recent holistic assessment report, which describes health sector performance across a range of indicators and provides the basis for wider sector dialogue on performance at the facility, district, regional and national level. |
| - Line 98. Some additional information in the background about the extent to which planning and implementation of malaria control policies are decentralized in Ghana would be helpful for context. The following paragraph (lines 99-117) focusses on data being reported up, but doesn’t elaborate on what kind of responses or decisions are made at the level of the CHPS. | We added more details to this paragraph in the background about how data are to be used at the CHPS level. Data allows CHPS staff to set their coverage targets, assess performance on key malaria indicators, and monitor malaria commodity stock levels to prevent stock-outs. |
| - Line 102. Are “monitoring charts to guide clinical meetings” individual patient charts, or aggregate case count numbers? | These monitoring charges present aggregated data for the month. We clarified in the text. |
| - Line 144. Facilities needed to meet both the error rate >30% AND the data completeness rate <90%? Should this be “or”? | The facilities that were prioritized had to meet both criteria, so it is correctly stated as “and”. |
| - Line 146. What kind of mistake generates an error flag? Did the validation exercise involve cross-referencing reported totals against OPD registers (presumably not since 605 facilities are mentioned later as the denominator)? It would also be helpful to know roughly how many indicators were validated – is this 3, 10, 100? | Errors were identified by verifying inconsistencies between data from the OPD registers, the monthly OPD Returns form and the national HMIS (DHIMS2).  The validation exercise to assess error included 15 indicators, listed and defined in an additional supplemental file (S1 Table):   1. Uncomplicated malaria suspected 2. Uncomplicated malaria suspected tested 3. Uncomplicated malaria tested positive 4. OPD attendants treated with Antimalarials 5. OPD attendants treated with ACTs 6. Number of suspected uncomplicated malaria cases tested for malaria parasites using RDT 7. Number of suspected uncomplicated malaria cases tested for malaria parasites using Microscopy 8. Number of suspected uncomplicated malaria cases tested positive using RDT 9. Number of suspected uncomplicated malaria cases tested positive using Microscopy 10. Number of ANC registrants 11. Number of pregnant women receiving IPTp1 12. Number of pregnant women receiving IPTp2 13. Number of pregnant women receiving IPTp3 14. Number of pregnant women receiving IPTp4 15. Number of pregnant women receiving IPTp5 |
| - Line 149. It is not clear how these three indicators relate to either the error rate of completeness rate. Were error rate and completeness only assessed for these indicators? | These three sentinel indicators were used to assess data completeness. A larger set of 15 indicators (above) were validated to assess error rate. We clarified this process and methods in the text. |
| - Lines 160. Should this be “all prioritized facilities”? | Yes, the reviewer is correct and we meant “all prioritized facilities.” We have changed the text accordingly. |
| - Line 203. “National DHIMS2 data” suggests that you used national aggregates of your indicators to evaluate the coaching program, which presumably was not the approach. | You are correct. We changed the text to indicate this was data collected at the facility and reported into DHIMS2. |
| - Lines 205-208. It is not clear how monthly data were handled to compare between the periods. Did you simply aggregate, or take the value for the month immediately before the next visit, or generate monthly means for each phase? | Aggregated monthly data were generated at the end of every month and a composite average calculated for all months in each period. We clarified this in the text. |
| - Lines 212-213. This statement implies that the facilities had to perform some additional data entry procedures for the evaluation on top of their usual responsibilities, which would bias any assessment of changes in data quality/completeness. Did the evaluation team actually download data from the DHIMS2 system? | All indicators were collected routinely and reported monthly to DHIMS2 by the Ghana Health Service SOP. There was no additional data collection burden on staff involved in the coaching visits. The evaluation team did not download any data from the national DHIMS2 system; all indicators used were routinely collected at the facility level and reported into the DHIMS2 system. We clarified terminology to describe the bounds of the data collected and reported. |
| - Line 225. I do not recall seeing specific dates when other phases were completed. How does this align with the overall implementation schedule? | Throughout the paper and in Figure 1, we added/clarified specific months and ranges of months of implementation of the different parts of the evaluation. |
| - Line 237. There has not been any previous explanation of how overall facility scores are generated. Are different components weighted? Is it a simple arithmetic sum of all questions with a “correct” response? | As the reviewer surmised, facility scores were generated by simple arithmetic with a “correct” or “yes” response. We calculated mean performance scores across all the facilities for each component. No components were weighted. We added this detail to the methods. |
| - Line 242. What are the case management quality indicators? To my knowledge, there are not any indicators in DHIMS2 that directly assess quality of case management since this can only be assessed by independent observation of consultations. | We considered a set of indicators as a case management cascade (Uncomplicated malaria cases suspected, Uncomplicated malaria cases tested, Uncomplicated malaria cases that tested positive/negative, and uncomplicated malaria cases treated with ACTs). These indicators help measure adherence to the T3 policy of malaria case management. We did not assess quality of individual consultations, but are using the skills and knowledge in conjunction with the case management cascade indicators to assess case management. We clarified this in the methods and the limitations paragraph. |
| - Lines 243. Please provide further details on how a paired t-test is conducted with data from 4 different periods. Are all comparisons made against the baseline or each each phase compared with the phase immediately prior? | Each phase was compared with the phase immediately prior. We clarified this in the text. |
| - Line 255. Is the facility-level DHIMS2 data truly publicly available? If so, this is very unusual but a fantastic resource for other researchers, and warrants inclusion of a link in the manuscript. Or do you mean it is available to authorized users only? | Facility-level DHIMS2 data are not publicly available but is available to authorized users or upon request from the GHS. We changed the description in line 255 to reflect accessibility more precisely. |
| - Line 256-257. It is unclear if these demographic details or coaching scores are included in the evaluation, and if so, how individual-level data have been summarized to facility-level. | There were no demographic details included in the knowledge and skills assessments, and all scores presented are aggregated. The only sociodemographic data on individuals that were collected was during the anonymous survey. We removed reference to demographic details and coaching from lines 256-257 to avoid confusing readers. |
| - Line 275-276. Was the change in individuals a result of staff turnover, or intentionally targeting different team members on each visit? | The change in individuals was not due to intentionally targeting different team members; rather, it reflected available staff on the day of the coaching visit (which also occasionally reflected new staff hires). We added this clarification to the text. |
| - Table 1. The interpretation of numbers in brackets is not explained in the column titles. | The numbers in brackets are 95% confidence intervals. We added this explanation to the table. |
| - Table 2. The contents of these five dimensions have not been presented elsewhere, which limits the ability to interpret (or assess the validity of) this table. | These five dimensions were explored using the standard USAID Data Quality Assurance Standard tool. The dimensions were mentioned, though not fully defined, in the methods. We added a reference to this tool as well as more details about these dimensions in the background. |
| - Table 3. Are the error rates the number of indicators that were not matching, or is this the empirical difference between a selected indicator in OPD registers and DHIMS2? | The error rate is the empirical difference between a selected indicator in OPD registers and DHIMS2. The Ghana Health Service standard is supposed to be +/- 10%. We added this definition to the manuscript. |
| - Figure 2 is low quality and looks to be a screenshot or similar. It may be worthwhile re-generating this figure in an alternative software. | We downloaded Figure 2 (which is an editable .tiff format we ran through the journal’s recommended PACE image checker) and did not find that it had low image quality. If there are continuing issues with this image, we are happy to revise it after the editorial team confirms the image does not have high enough resolution. |
| - The supplementary figure resolution means that the small font text is unreadable. While it’s nice to see an example that’s been completed, you may consider also including a copy of the template where all the text is readable. | Unfortunately, we were unable to obtain this template in a higher resolution because the project has ended and the study data files are no longer available. |
